# Supplementary figures and images for: Exome-wide association study to identify rare variants influencing COVID-19 outcomes: Results from the Host Genetics Initiative
Source: PLoS Genet. 2022 Nov 3;18(11):e1010367. doi: 10.1371/journal.pgen.1010367 (PMC9632827; doi:10.1371/journal.pgen.1010367)

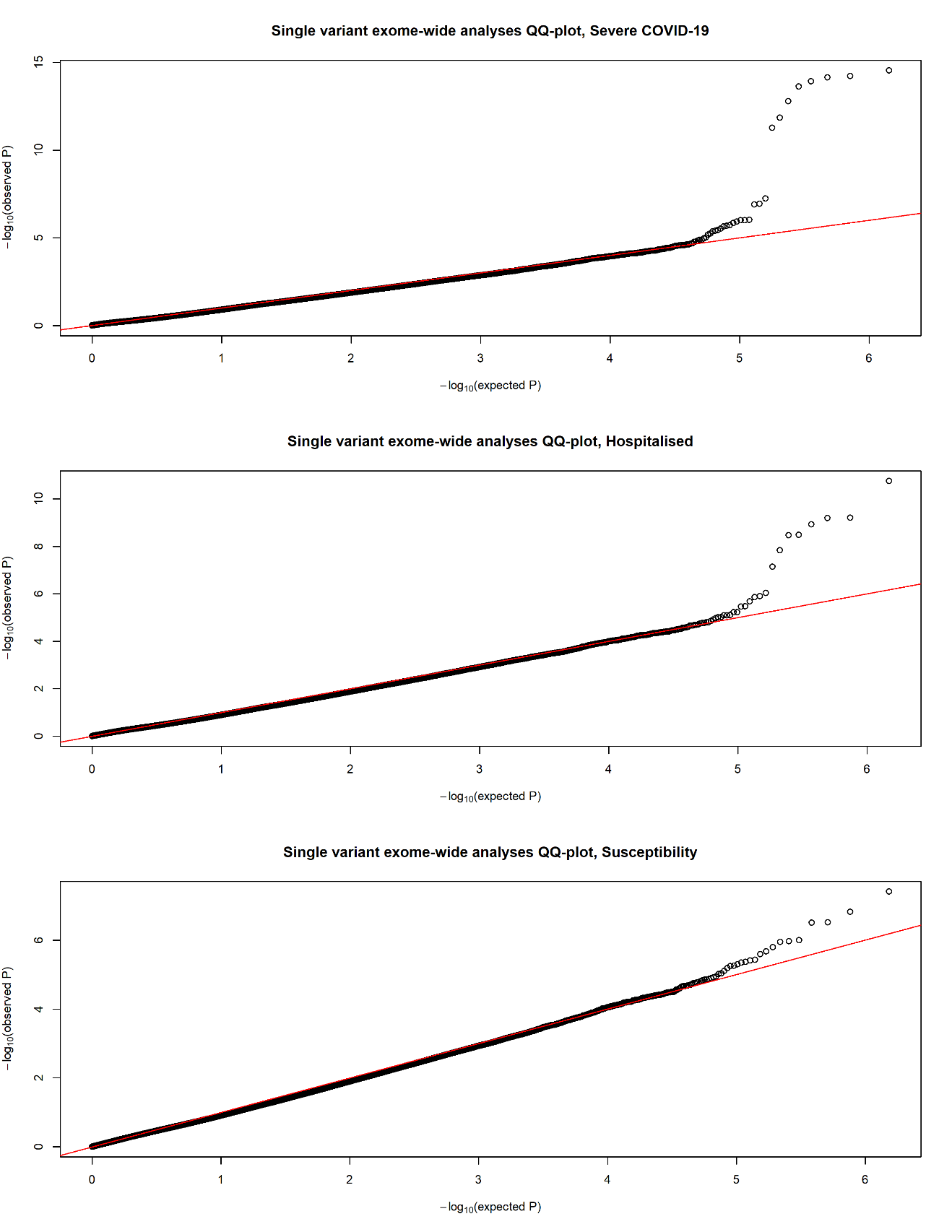


QQ plot and Manhattan plot for the exome-wide single variant association studies.

Supplement: S1 Fig — (DOCX) [file pgen.1010367.s010.docx]

**
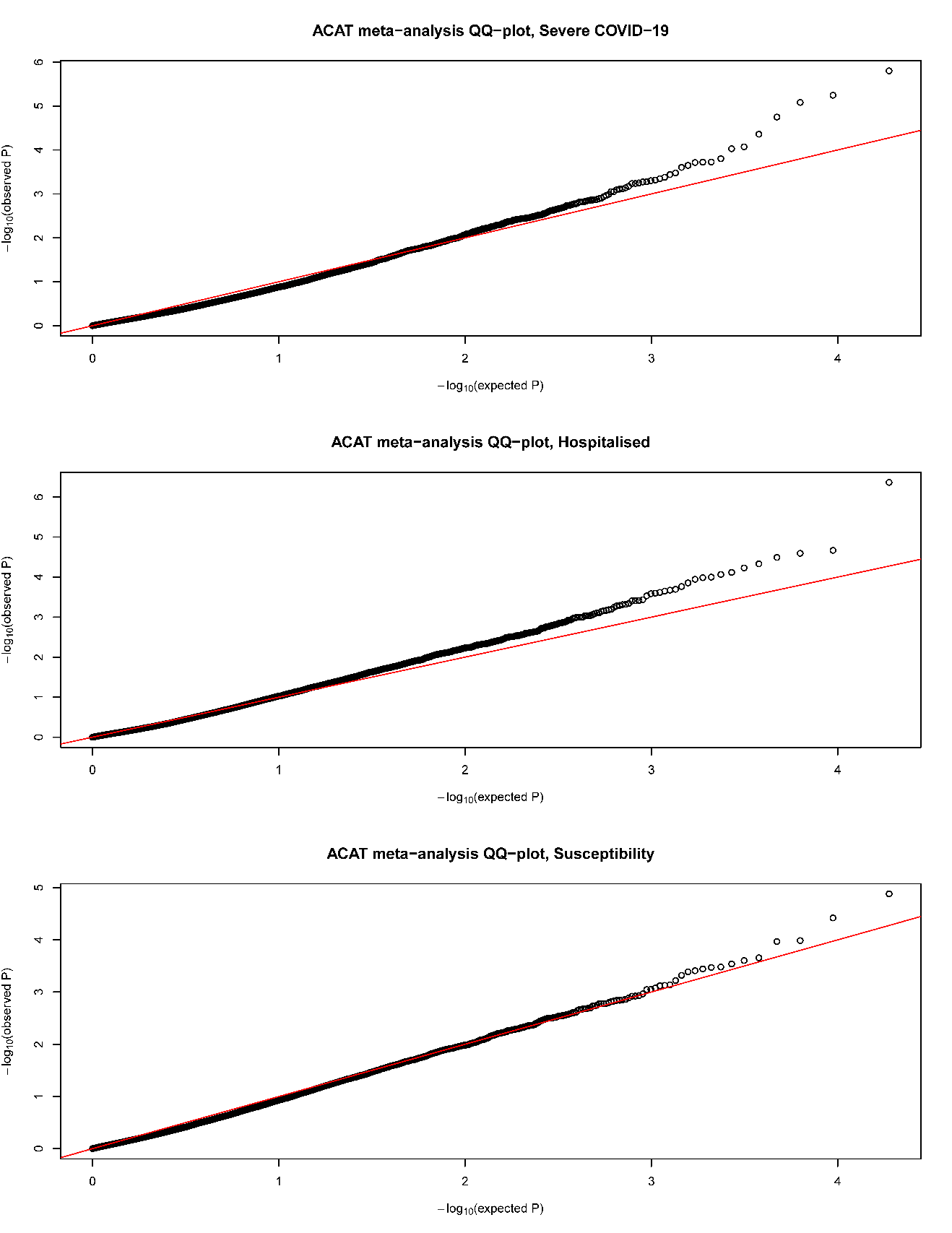
**

QQ plot from exome burden test ACAT meta-analyses.

Supplement: S6 Fig — (DOCX) [file pgen.1010367.s015.docx]
