# Supplementary material for: Exome-wide association study to identify rare variants influencing COVID-19 outcomes: Results from the Host Genetics Initiative
Source: PLoS Genet. 2022 Nov 3;18(11):e1010367. doi: 10.1371/journal.pgen.1010367 (PMC9632827; doi:10.1371/journal.pgen.1010367)
Supplement: S2 Fig — Note that all ancestries are shown together. Black dashed line represents the nominal statistical significance threshold (p = 0.05). (DOCX) [file pgen.1010367.s011.docx]

Chromosome 3 exome single variant association studies result by cohort for the severe disease phenotype. Note that all ancestries are shown together. Black dashed line represents the nominal statistical significance threshold (p=0.05).
