# Supplementary material for: Exome-wide association study to identify rare variants influencing COVID-19 outcomes: Results from the Host Genetics Initiative
Source: PLoS Genet. 2022 Nov 3;18(11):e1010367. doi: 10.1371/journal.pgen.1010367 (PMC9632827; doi:10.1371/journal.pgen.1010367)
Supplement: S5 Fig — (DOCX) [file pgen.1010367.s014.docx]

**

**Single variant association study results at the three novel loci (OR and 95% CI).
