# Supplementary material for: Exome-wide association study to identify rare variants influencing COVID-19 outcomes: Results from the Host Genetics Initiative
Source: PLoS Genet. 2022 Nov 3;18(11):e1010367. doi: 10.1371/journal.pgen.1010367 (PMC9632827; doi:10.1371/journal.pgen.1010367)
Supplement: S10 Fig — X-axes are cut at -10 and 10. (DOCX) [file pgen.1010367.s019.docx]

*MARK1* beta coefficients (on logistic scale) with 95% interval for the severe COVID-19 phenotype, MAF<1%. X-axes are cut at -10 and 10.
