# Supplementary material for: Exome-wide association study to identify rare variants influencing COVID-19 outcomes: Results from the Host Genetics Initiative
Source: PLoS Genet. 2022 Nov 3;18(11):e1010367. doi: 10.1371/journal.pgen.1010367 (PMC9632827; doi:10.1371/journal.pgen.1010367)
Supplement: S13 Fig — X-axis cut at 50. Figures show odds ratios and 95% confidence intervals. (DOCX) [file pgen.1010367.s022.docx]

Ancestry stratified results for *TLR7*, *MARK1*, and *ABO*. X-axis cut at 50. Figures show odds ratios and 95% confidence intervals
